# ACP3 Theranostics in Prostate Cancer

**DOI:** 10.3390/cancers18213511

**Published:** 2026-10-31

**Authors:** Zhilin Luo, Danlei Chen, Tianyi Ma, Pingping Zeng, Xuejie Li, Haoyuan Shi, Zhiyuan Hu

**Affiliations:** 1 School of Chemical Engineering and Pharmacy, Wuhan Institute of Technology, Wuhan 430205, China; luozl2025@nanoctr.cn; 2 CAS Key Laboratory for Biomedical Effects of Nanomaterials and Nanosafety, CAS Key Laboratory of Standardization and Measurement for Nanotechnology, CAS Center for Excellence in Nanoscience, National Center for Nanoscience and Technology, Beijing 100190, China; chendanlei25@mails.ucas.ac.cn (D.C.); maty2025@nanoctr.cn (T.M.); zengpp2025@nanoctr.cn (P.Z.); lixj2024@nanoctr.cn (X.L.); 3 Fujian Provincial Key Laboratory of Brain Aging and Neurodegenerative Diseases, School of Basic Medical Sciences, Fujian Medical University, Fuzhou 350122, China; 4 School of Future Medicine, Beijing University of Chinese Medicine, Beijing 100190, China

**Keywords:** ACP3, prostate cancer, PSMA, molecular imaging, radioligand therapy, theranostics

## Abstract

Prostate-specific membrane antigen (PSMA)-targeted positron emission tomography (PET) and radioligand therapy have established a major clinical paradigm for prostate cancer theranostics. Their performance is influenced by interpatient and interlesional heterogeneity, treatment-related changes in PSMA expression, and physiological ligand uptake in organs such as the salivary glands and kidneys, creating a rationale for complementary targets with different lesion coverage and normal-tissue distribution. ACP3 (acid phosphatase 3; historical symbol ACPP) encodes prostatic acid phosphatase (PAP), which is also termed prostate-specific acid phosphatase (PSAP) in older pathology literature. PAP has served as a serum biomarker, an immunohistochemical marker of prostatic origin, and an immunotherapeutic antigen. DNA-encoded library screening identified the high-affinity PAP ligand OncoACP3, and the resulting [^177^Lu]Lu-OncoACP3-DOTAGA radioligand and OncoACP3-GlyPro-MMAE small-molecule drug conjugate achieved target-dependent payload delivery and antitumor activity in preclinical models. First-in-human PET and subsequent pharmacokinetic and diagnostic dosimetry studies of [^68^Ga]Ga-OncoACP3-DOTA demonstrated visualization of localized and metastatic prostate cancer lesions and a normal-organ distribution distinct from that of PSMA-targeted ligands. This narrative review synthesizes the molecular characteristics and biological functions of PAP, historical clinical applications, and modern ACP3-targeted imaging and therapeutic platforms. Existing studies now provide a biological foundation, preclinical proof of targeted payload delivery, and early human imaging, pharmacokinetic, and diagnostic-dosimetry evidence. Prospective development should next define the ligand-accessible PAP pool, imaging-positivity criteria, therapeutic dosimetry, and the patient groups in whom ACP3 adds value to the PSMA theranostic framework.

## Simple Summary

Prostate-specific membrane antigen has transformed molecular imaging and radioligand therapy for prostate cancer, but heterogeneous target expression and uptake in dose-limiting normal organs leave room for complementary targets. Acid phosphatase 3 encodes prostatic acid phosphatase, a protein with a long clinical history as a serum marker, pathological marker, and immunotherapeutic antigen. The discovery of the high-affinity ligand OncoACP3 enabled targeted delivery of imaging and therapeutic payloads. Preclinical studies demonstrated selective tumor localization and antitumor activity, whereas early human positron emission tomography studies showed that ACP3-targeted imaging can visualize prostate cancer lesions and has a biodistribution pattern that differs from that of prostate-specific membrane antigen ligands. This review evaluates the biological rationale and current evidence for ACP3-directed theranostics. It also defines the evidence still needed before clinical utility can be established, including identification of the ligand-accessible protein pool, standardized imaging criteria, therapeutic dosimetry, and prospective validation in patients whose disease is insufficiently characterized or treated by existing prostate-specific membrane antigen strategies.

## 1. Introduction

Prostate cancer is a common malignancy among men worldwide, with approximately 1.468 million new cases and 397,000 deaths estimated in 2022 [1]. Although localized disease can often be controlled by surgery or radiotherapy, recurrent and metastatic disease remain heterogeneous clinical challenges [2]. Androgen deprivation, androgen receptor pathway inhibitors (ARPIs), taxane chemotherapy, radium-223, and immunotherapy have expanded treatment options across different disease settings [3,4,5,6,7,8,9,10]. However, clonal evolution, lineage plasticity, and acquired resistance can limit durable benefit. Heterogeneity occurs between patients, among lesions within the same patient, and within individual tumors; treatment may further alter differentiation and target expression [11,12,13,14,15,16,17]. Consequently, target-based treatment requires assessment of disease distribution, prior treatment, and ligand-accessible target expression. Theranostic strategies address these requirements by linking whole-body imaging, patient selection, dose assessment, and response monitoring through a shared molecular-recognition framework [18]. This framework is particularly relevant when a target’s presence in a tissue sample does not adequately represent its accessibility or distribution across the patient’s disease.

Prostate-specific membrane antigen (PSMA) provides the established clinical benchmark for prostate cancer theranostics, with evidence from prospective positron emission tomography (PET) studies and randomized radioligand-therapy trials [19,20,21,22,23,24,25]. The U.S. Food and Drug Administration (FDA) expanded the indication for [^177^Lu]Lu-PSMA-617 in 2025 to include patients with PSMA-positive metastatic castration-resistant prostate cancer (mCRPC) previously treated with an ARPI who were considered appropriate to delay taxane chemotherapy [26]. In July 2026, the FDA approved its combination with an ARPI for PSMA-positive metastatic androgen pathway modulation-naïve or -sensitive prostate cancer [27]. These developments illustrate the progression of PSMA-targeted therapy toward earlier metastatic disease and provide a clinical reference for evaluating emerging theranostic targets.

Nevertheless, PSMA expression varies with differentiation, treatment exposure, and neuroendocrine transformation, and some active lesions exhibit insufficient uptake for PSMA-directed approaches [12,16,28,29,30,31,32,33,34]. Physiological uptake in normal organs, including the salivary glands and kidneys, also influences the therapeutic window [35,36,37,38]. These limitations motivate investigation of complementary targets with different lesion coverage and normal-tissue distribution. Such development requires more than demonstration of target abundance: a candidate ligand must reach its target in vivo, remain sufficiently retained, and support imaging or payload delivery. The biological and clinical evidence needed to establish these properties therefore provides the framework for assessing ACP3.

ACP3 encodes prostatic acid phosphatase (PAP), which has served as a serum biomarker, an immunohistochemical marker of prostatic origin, and an immunotherapeutic antigen [9,39]. Discovery of the high-affinity ligand OncoACP3 extended this history to small-molecule targeting, preclinical payload delivery, and early human PET imaging and diagnostic dosimetry [40,41,42,43]. These developments connect several distinct levels of evidence: circulating protein, tissue expression, antigenicity, and in vivo ligand accessibility. Although the historical PAP literature provides a substantial biological foundation, its implications for direct ligand targeting require separate assessment. In particular, tissue positivity and immune recognition do not themselves establish accessibility to a systemically administered ligand.

Existing reviews and commentaries have described PAP biology and the emergence of ACP3-targeted imaging and theranostic strategies [44,45,46,47]. Building on these contributions, this narrative review summarizes the current evidence for ACP3-targeted strategies and discusses their potential role as a clinically useful complement to PSMA. Its emphasis is on distinguishing PAP expression from ligand accessibility, and preclinical activity and human imaging feasibility from demonstrated clinical utility. Historical biomarker and immunotherapy findings are considered alongside ligand-based studies, with their different mechanisms and evidentiary implications explicitly separated. The review addresses four linked questions: which PAP pool is accessible to systemically administered ligands; how accessibility varies across disease settings; whether ACP3-targeted imaging provides validated, clinically relevant information beyond PSMA; and what construct-specific pharmacological, dosimetric, and clinical evidence is required for therapeutic development. This framework connects target biology with the studies needed to evaluate patient selection, safety, and clinical benefit.

### Literature Search and Evidence Selection

This narrative review was informed by literature searches in PubMed/MEDLINE and consultation of publicly available regulatory sources. This review included relevant literature published online or in print on or before 1 October 2026. Publications first available online or in print on or before this date were eligible for inclusion. Search terms included “ACP3”, “ACPP”, “prostatic acid phosphatase”, “PAP”, “PSAP”, and “OncoACP3”, used individually or in combination with “prostate cancer”, “positron emission tomography”, “molecular imaging”, “radioligand therapy”, and “theranostics”. Reference lists of key publications were also examined to identify additional relevant studies. Publications were selected for their relevance to PAP biology, historical clinical applications, ligand development, molecular imaging, and therapeutic payload delivery. Seminal studies were retained to provide historical and biological context, while recent original studies were prioritized when describing ACP3-targeted platform development. Original human studies were prioritized for clinical interpretation, whereas preclinical findings were distinguished according to biochemical, cellular, and animal evidence.

## 2. Biological Basis of ACP3 Targeting

### 2.1. Nomenclature, Molecular Forms, and Subcellular Localization

**Figure 1:**
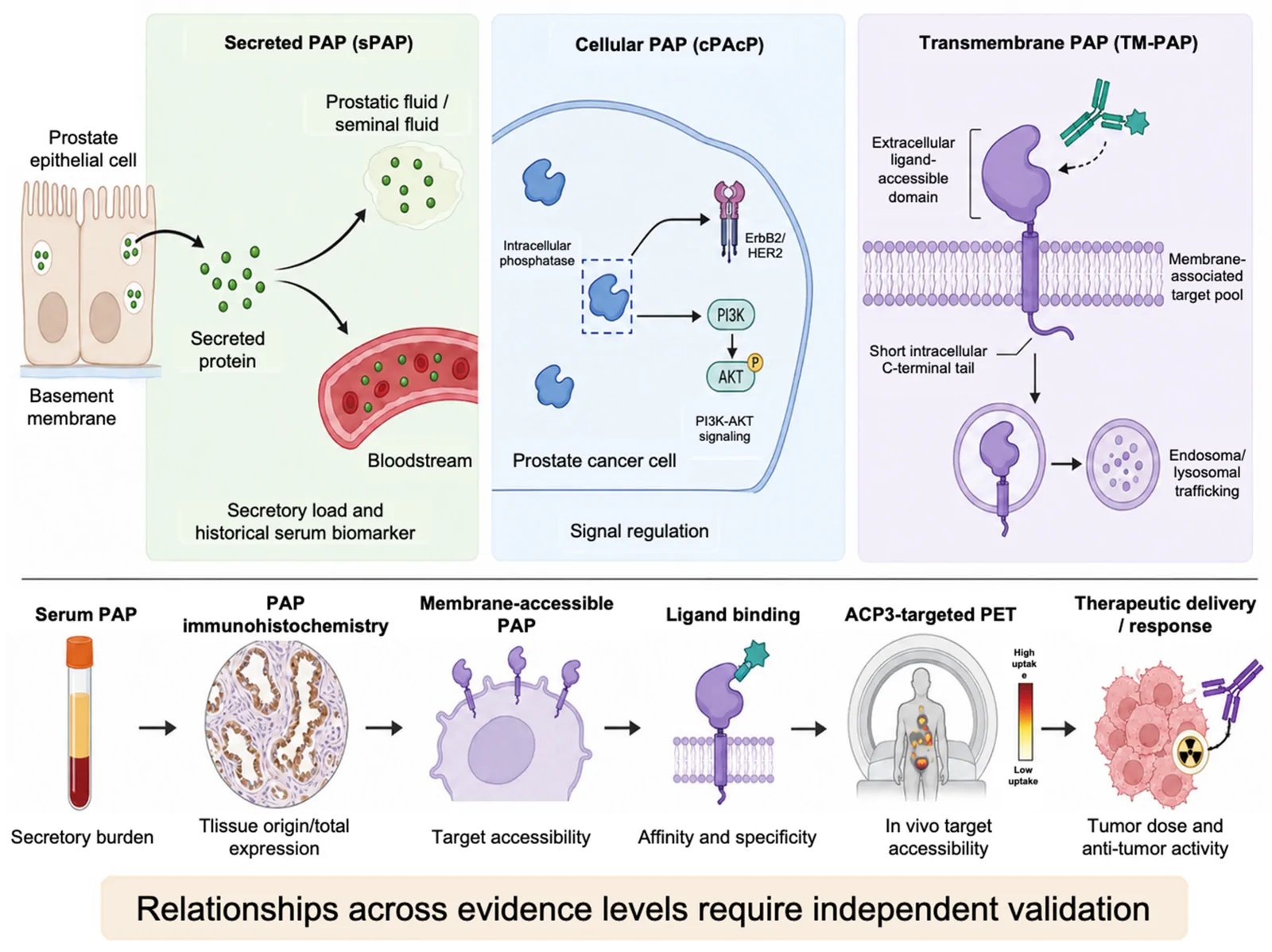
Major molecular forms and subcellular localizations of PAP and different levels of evidence for target validation.

ACP3 (historical symbol ACPP) denotes the gene encoding prostatic acid phosphatase (PAP); PAcP and PSAP are historical terms used for the protein in parts of the literature [48,49,50,51]. Throughout this review, ACP3 is used for gene or RNA measurements, whereas PAP is used for protein abundance, localization, and biochemical activity. Historical terminology is retained where necessary to describe the original studies. The established names OncoACP3 and “ACP3-targeted” are retained; the latter refers to targeting of the ACP3-encoded PAP protein rather than the gene. Human PAP is a dimeric glycoprotein with acid phosphatase activity [52,53]. Secretory PAP, intracellular/cellular PAP (cPAcP), and transmembrane PAP (TM-PAP) have distinct reported localizations and functions (Table 1; Figure 1) [53,54,55]. Here, “ligand-accessible PAP” denotes the PAP pool available for binding by a particular ligand under the relevant biological and delivery conditions. It is not synonymous with total tissue PAP or restricted by definition to membrane-associated PAP. Protein expression, ligand binding, and PET uptake are therefore described separately according to the measurement performed.

Biochemical and crystallographic studies characterize PAP as a glycosylated homodimer with a defined catalytic pocket [52,56]. Recombinant-expression and active-site mutagenesis experiments further characterized its enzymatic properties. OncoACP3 was identified by screening against purified PAP, and the lead series showed subnanomolar enzymatic inhibition, nanomolar binding in orthogonal assays, and slow dissociation. Cell-binding, competition, tissue-autoradiography, and in vivo blocking experiments further connected biochemical activity with target-dependent recognition in cells, native tissue, and tumor models [40].

Studies differ in their descriptions of the subcellular localization and functional forms of PAP. Chuang et al. characterized cPAcP in prostate cancer cell models as an intracellular PAP with protein tyrosine phosphatase activity and reported dephosphorylation of ErbB-2; Muniyan et al. subsequently summarized the intracellular functions of cPAcP in tumor suppression and related signaling [54,57,58]. By contrast, Quintero et al. reported that TM-PAP localized predominantly to the plasma membrane–endosome–lysosome pathway in their model and did not detect a distinct cytosolic PAP pool [59]. These differences may be related to the splice variants studied, antibodies, cell models, and detection methods. Accordingly, subsequent theranostic development should define the PAP form, binding site, and cellular compartment recognized by OncoACP3 rather than rely on the undifferentiated label “ACP3-positive”.

The proportions of the secretory, intracellular, and transmembrane forms may also change with cell differentiation and disease progression. For diagnostic tracers, secreted proteins accessible from the vascular side, extracellular matrix-bound proteins, or membrane proteins may all contribute to the tumor signal. For cytotoxic payloads, therapeutic activity also depends on linker stability, tumor-localized payload release, and uptake of the released drug by target cells [40,60,61]. Radionuclide constructs can additionally irradiate nearby target-negative cells through cross-fire effects. Each payload class should therefore be optimized and evaluated separately with respect to target-density requirements, pharmacokinetics, and therapeutic index.

**Table 1:** PAP molecular forms and their relevance to ACP3-targeted theranostics.

Molecular Form	Principal Localization/Structural Characteristics	Uses of Existing Evidence	Significance for Theranostic Development	Key Limitations	Reference(s)
Secretory PAP (sPAP)	Secreted by prostate epithelial cells; can enter prostatic fluid, semen, and the circulation	Historical serum marker; disease burden-related studies; antigenic basis for PAP-targeted immunotherapy	Supports research on serum/body-fluid markers and PAP-related immunotherapy	Does not establish the accessibility of tumor-associated PAP to a circulating ligand	[9,62,63,64,65,66]
Intracellular/cell-associated PAP (cPAcP)	Located mainly intracellularly, with protein tyrosine phosphatase-related activity	Studies of ErbB2/HER2 dephosphorylation, PI3K–AKT-related signaling, and functions in tumor biology	Helps elucidate the relationships of PAP with prostate epithelial differentiation, cell proliferation, and disease progression	Primarily reflects intracellular PAP biology.	[54,57,58]
Transmembrane PAP (TM-PAP)	Type I transmembrane protein; has an extracellular N-terminal phosphatase domain and an intracellular C-terminus and is associated with endosomal/lysosomal trafficking	Studies of membrane localization, internalization/trafficking, antibodies, and other membrane-targeting platforms	Provides a biological basis for antibody or ligand recognition of membrane-associated PAP	Whether TM-PAP is the predominant PAP form recognized by OncoACP3 in patients remains to be established.	[49,55,59,67,68]

### 2.2. Tissue Expression Heterogeneity and Normal Tissue Boundaries

The Human Protein Atlas classifies ACP3 RNA as having prostate tissue-enriched expression, and traditional pathology has also long regarded PAP—termed PSAP in portions of the historical literature—as a marker of prostate origin [69,70,71]. Tribian et al. conducted a large-scale tissue-microarray study that included 17,747 prostate cancer cases, of which 15,455 had evaluable PSAP immunohistochemistry results; 96.9% of the evaluable cases were PSAP-positive [69]. This percentage describes tissue immunoreactivity in the analyzed cohort and should not be interpreted as the proportion of patients eligible for OncoACP3-based imaging or therapy. Across different pathological subtypes, the PSAP positivity rates for Gleason 3 + 3, Gleason 4 + 4, recurrent carcinoma after androgen-deprivation therapy, Gleason 5 + 5, and small-cell neuroendocrine carcinoma were 100%, 95.5%, 93.8%, 91.0%, and 31.2%, respectively [69]. These results establish frequent retention of PAP protein in prostate adenocarcinoma and a marked decline in small-cell neuroendocrine carcinoma. Paired imaging–pathology studies should next determine how this differentiation-associated pattern overlaps with PSMA-low and PSMA-negative lesions.

The high frequency of PAP immunoreactivity provides a biological rationale for investigating PAP-directed targeting. However, routine immunohistochemistry does not establish whether the detected protein pool is accessible to systemically administered OncoACP3. This relationship requires paired assessment of staining intensity, the proportion of positive cells, protein localization, and interlesional heterogeneity, together with ex vivo ligand binding and lesion-level PET uptake. Such studies could determine whether, and under what conditions, PAP immunohistochemistry predicts ligand binding and in vivo uptake, rather than assuming that tissue positivity establishes suitability for imaging or treatment.

Human imaging now provides direct normal-organ distribution data for [^68^Ga]Ga-OncoACP3-DOTA. In seven patients, the tracer showed dual renal and hepatobiliary clearance, a mean effective dose of 0.0192 mSv/MBq, and absorbed-dose coefficients of 0.0913 mGy/MBq for red marrow, 0.0657 mGy/MBq for the kidneys, 0.0563 mGy/MBq for the liver, and 0.0135 mGy/MBq for the salivary glands [42]. These findings characterize organ exposure to the diagnostic [^68^Ga]Ga-OncoACP3-DOTA construct; their implications for therapeutic development are discussed in Section 4.3 and Section 6.3. Normal-organ uptake may reflect PAP-mediated binding, off-target interactions, or tracer distribution and clearance; PET uptake alone does not distinguish these mechanisms. These possibilities require construct-specific binding and biodistribution studies.

The diagnostic biodistribution findings define the organs and measurements to prioritize as ACP3-targeted therapeutics advance. Longer-lived radionuclide constructs should undergo serial time–activity measurement and organ-specific dosimetry across planned treatment cycles [37]. MMAE and other cytotoxic conjugates should be evaluated for linker stability, circulating free payload, tumor-selective release, and cumulative systemic toxicity [40,60,61].

### 2.3. Biological Functions and Disease Progression

Chuang et al. showed that cPAcP dephosphorylates ErbB-2 Tyr1221/1222 and modulates downstream Shc, ERK, AKT, and STAT signaling and cell proliferation; Muniyan et al. linked reduced cPAcP expression to androgen-independent growth and greater tumorigenicity [54,57,58]. In a non-tumor preclinical system, Zylka et al. demonstrated that PAP functions as an ecto-5’-nucleotidase that converts AMP to adenosine [72]. Together, these findings position PAP as a regulator of differentiation, receptor signaling, and extracellular adenosine generation and provide a mechanistic framework for human validation [73]. Longitudinal studies that map PAP and PSMA in the same patients and lesions can test how AR remodeling [74,75], genomic alterations, and lineage plasticity reshape both targets during treatment [13,15,16,28].

The association between lower PAP expression and more aggressive pathological phenotypes suggests that PAP may be preferentially retained in better-differentiated adenocarcinoma, whereas expression may be reduced in some poorly differentiated or neuroendocrine lesions [69]. Kirschenbaum et al. reported in a 2024 preclinical study that TM-PAP remained expressed in VCaP and enzalutamide-resistant models and could be upregulated by hypoxia [68]. Taken together, these findings indicate that PAP expression may be jointly influenced by tumor lineage, treatment background, and the microenvironment, and its distribution in advanced resistant disease needs to be interpreted at the patient and lesion levels.

TM-PAP-mediated conversion of AMP to adenosine provides a testable link between PAP biology and the tumor microenvironment [68,72]. Measuring local adenosine production together with immune-cell states in human tumors would determine whether this pathway contributes to PAP-associated tumor biology or treatment response.

### 2.4. From Target Expression to Clinical Exploitability

For a molecular target to support theranostic development, four features are particularly relevant: sufficient contrast between tumor and critical normal tissues, vascular access and specific ligand binding, adequate tumor retention after binding, and sufficient target coverage across clinically relevant lesions. Available studies document frequent PAP tissue expression, high-affinity OncoACP3 recognition, target-dependent tumor uptake, and early human PET visualization [40,41,69]. The next step is to integrate these dimensions within the same patient cohort by correlating tissue expression and ex vivo ligand binding with lesion-level uptake, whole-body lesion coverage, and normal-organ dosimetry.

Secretory and membrane-associated PAP may contribute differently to ligand distribution and retention. Tumor-interstitial PAP could support local retention, whereas circulating PAP could alter blood-pool kinetics [63]. Prospective studies should therefore measure serum PAP, ligand mass, injected activity, and lesion uptake together to test for an antigen-sink effect and define any mass-dependent pharmacokinetics.

Imaging and therapy place different demands on target coverage. A tracer can be clinically useful if it identifies additional validated lesions that alter staging or management, whereas systemic monotherapy requires adequate absorbed dose across the lesions that determine outcome. For ACP3, this distinction should be tested by comparing lesion-level detection with predicted or measured therapeutic dose rather than assuming that PET visibility alone establishes therapeutic suitability.

Serial imaging during treatment can determine whether the distribution of uptake on ACP3-targeted PET remains stable across cycles and whether it changes independently of PSMA. If longitudinal studies demonstrate reproducible and partially nonoverlapping patterns, they would provide a rationale for evaluating treatment sequencing or dual-target strategies, with biopsies of selected progressive lesions used for mechanistic correlation.

Taken together, current evidence establishes PAP as a biologically plausible and ligand-accessible candidate target and supports continued development of ACP3-targeted imaging and payload delivery. Clinical exploitability can now be defined through four linked measurements: ligand-accessible PAP, whole-body lesion coverage, normal-tissue exposure, and target stability over time.

## 3. Historical Trajectory of Clinical Evidence: Biomarker, Pathological Marker, and Immunologic Antigen

### 3.1. Serum PAP: Historical Value and Modern Boundaries

**Figure 2:**
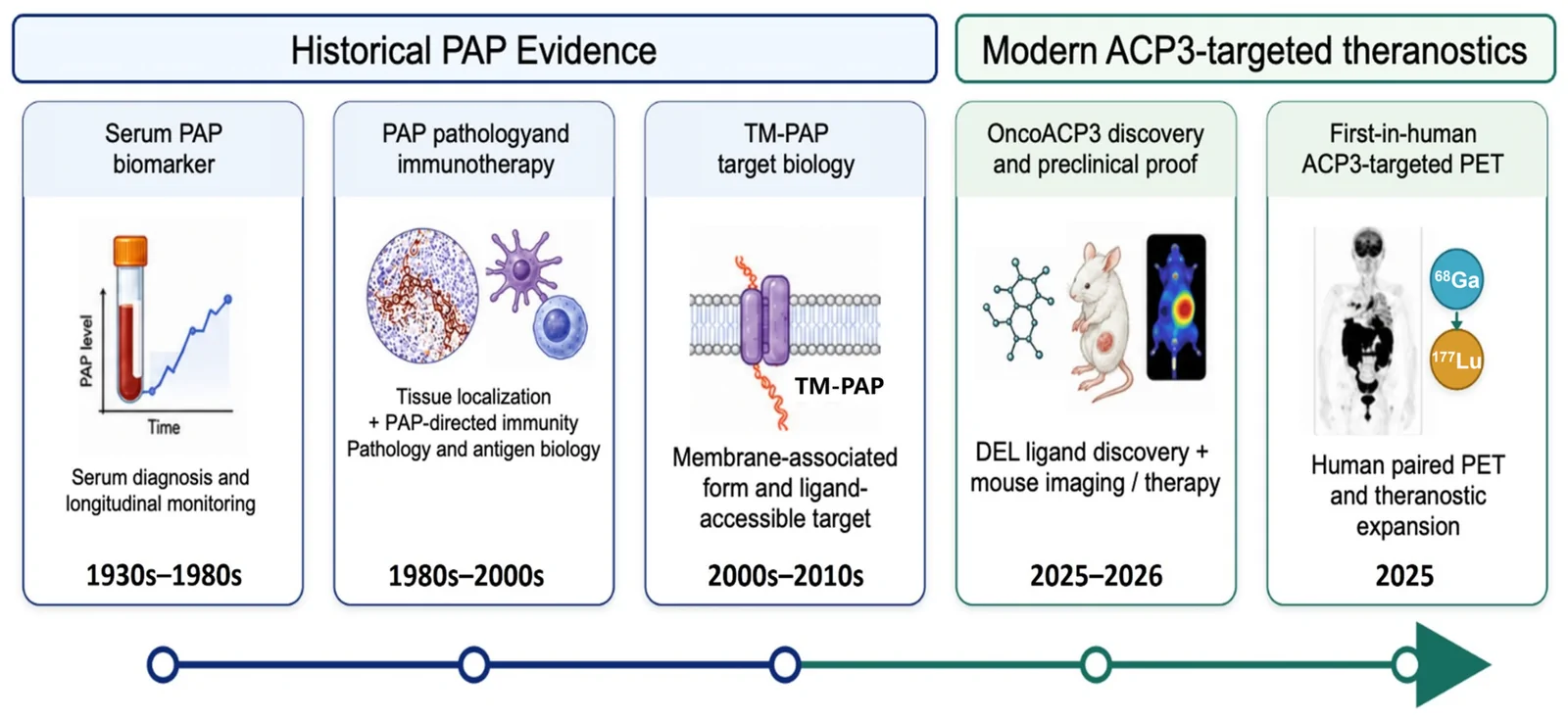
Historical development of PAP research and the emergence of ACP3-targeted theranostics. Blue denotes historical PAP evidence, whereas green denotes modern ligand-based preclinical and early human imaging studies; the bottom arrow indicates chronological progression. The ^68^Ga-to-^177^Lu arrow illustrates the proposed translation from diagnostic imaging to therapeutic development and does not indicate established ACP3-targeted therapy in humans.

In 1938, Alexander B. Gutman and Ethel Benedict Gutman reported elevated serum acid phosphatase in patients with metastatic prostate cancer, helping to establish PAP as one of the earliest clinically used tumor markers (Figure 2; Table 2) [39]. After prostate-specific antigen (PSA) became widely used, PAP fell out of routine screening because of its lower sensitivity in early disease; comparative studies showed that the sensitivity of PSA for both newly diagnosed and recurrent disease was higher than that of PAP [64,76]. Two intermediate- to high-risk cohorts receiving radiotherapy or brachytherapy suggested that pretreatment PAP may be associated with distant failure or cancer-specific survival [65,66]. Most of these historical data are retrospective and heterogeneous in assay platform and treatment context; they support an association of serum PAP with disease burden or differentiation.

Serum PAP may capture aspects of disease burden that differ from those reflected by PSA, providing a rationale to test its incremental prognostic value alongside stage, grade, and contemporary imaging [66,76].

For modern ACP3 imaging, serum PAP represents a testable pharmacokinetic variable: circulating PAP could act as a soluble antigen sink and alter blood clearance, tumor delivery, or dosimetry. Prospective studies should analyze serum PAP together with ligand mass, injected activity, blood time–activity curves, lesion SUV, and normal-organ uptake to determine whether this effect is saturable and whether it contributes to interpatient variation.

### 3.2. PAP Immunohistochemistry: Determination of Origin and Expression Stratification

PAP immunohistochemistry, termed PSAP immunohistochemistry in portions of the pathology literature, can be used together with PSA and NKX3.1 to support determination of prostatic origin in metastatic lesions [69,70]. Of particular relevance to this review, the tissue-microarray study by Tribian et al. also demonstrated pathological heterogeneity in PAP protein expression: most prostate adenocarcinomas retained a high positivity rate, whereas the positivity rate was markedly lower in small-cell neuroendocrine carcinoma. Correlating lesion SUV with PAP staining intensity, isoform-specific localization, RNA in situ hybridization, and ex vivo ligand binding in contemporaneous biopsies could help define the ligand-accessible PAP pool represented by the imaging signal.

In metastatic adenocarcinoma of unknown origin, negativity for a single marker is insufficient to exclude a prostatic origin, particularly after dedifferentiation, endocrine therapy, or neuroendocrine transformation. A combined evaluation of PAP/PSAP, PSA, and NKX3.1 can reduce misclassification caused by loss of an individual marker [69,70]. A similar caution applies to molecular imaging: a negative ACP3-targeted PET result may reflect low ligand-accessible PAP, but it may also result from lesion size, perfusion, necrosis, pharmacokinetics, or imaging time. Correlative analyses of multimodal imaging, histology, and follow-up outcomes may help distinguish biological negativity from technical false-negative findings.

Longitudinal assessment is the next priority for defining treatment-related changes in PAP. Serial ACP3-targeted and PSMA PET before and after AR-pathway inhibition, chemotherapy, or radioligand therapy can provide whole-body maps of target redistribution, while biopsies of accessible concordant and discordant lesions can establish the corresponding changes in PAP protein expression.

### 3.3. PAP-Directed Immunotherapy and Its Relevance to Ligand Targeting

Sipuleucel-T is an autologous cellular immunotherapy prepared using patient-derived antigen-presenting cells activated ex vivo with PA2024, a PAP–granulocyte–macrophage colony-stimulating factor fusion protein [77]. In the phase III IMPACT trial involving 512 patients with mCRPC, median overall survival was 25.8 months with sipuleucel-T versus 21.7 months with control treatment (hazard ratio for death, 0.78), whereas time to objective disease progression did not differ significantly [9]. These findings establish PAP as a clinically actionable immunologic antigen [78,79].

PAP DNA vaccines have also induced PAP-specific immune responses [80,81,82,83,84]. However, a randomized phase II trial involving 99 patients found no significant between-group difference in the prespecified 2-year metastasis-free survival endpoint [85]. These studies support PAP immunogenicity while underscoring the need to distinguish immune activation from demonstrated clinical benefit.

PAP-directed vaccination depends on antigen processing, presentation, and downstream immune responses [86,87,88,89,90,91]. In contrast, direct ligand targeting requires an administered ligand to reach and bind an accessible PAP protein pool, with sufficient tumor retention and payload delivery for the intended application. Successful PAP-directed immunotherapy therefore does not establish the accessibility of PAP to OncoACP3 or its suitability for radioligand targeting. Ligand binding, tumor delivery, absorbed doses, and treatment safety and efficacy require independent, platform-specific validation. The immunotherapy literature thus provides biological and clinical context for ACP3-targeted development without establishing the feasibility of ligand-based therapy [86,87,88,92].

**Table 2:** Representative Evidence and Key Milestones in the Evolution of PAP Research and ACP3 Target Development.

Stage/Representative Study	Study Population or Technology Platform	Main Findings	Current Evidentiary Support	Current Interpretation	Reference(s)
Gutman et al., 1938 [39]	Serum acid phosphatase in patients with metastatic prostate cancer	Elevated serum acid phosphatase in patients with metastatic prostate cancer	Established an early clinical association between elevated serum acid phosphatase and metastatic prostate cancer	Historical serum-biomarker evidence.	[39]
PAP Research in the Post-PSA Era	Comparative Serum Testing and Prognostic Studies of PSA and PAP	PSA gradually replaced PAP for routine screening and monitoring; PAP remained associated with some measures of advanced disease burden and prognostic indicators	PAP retained value as a prostate-associated marker and for research on disease burden	Supports disease-burden and prognostic research.	[64,65,66,76]
Pathology Studies of PSAP	Large-Sample Tissue Microarrays and Immunohistochemistry	Most prostate adenocarcinomas retain PSAP expression; expression decreases in poorly differentiated and small-cell neuroendocrine carcinomas	PSAP is highly informative for identifying prostatic tissue lineage and indicates that expression is influenced by tumor differentiation status	Supports prostate-lineage identification and PAP expression profiling; does not establish ligand accessibility.	[69,70]
sipuleucel-T/IMPACT	Autologous cellular immunotherapy for mCRPC; PAP-GM-CSF antigen system	A phase III study showed an overall survival benefit with sipuleucel-T	Provides phase III evidence that PAP-directed autologous cellular immunotherapy produces clinical benefit	Establishes PAP as a clinically actionable immunologic antigen.	[9]
Immunotherapy Studies Including PAP DNA Vaccines	PAP-Encoding DNA Vaccines and Immunogenicity Studies	Can induce PAP-related immune responses and demonstrates early clinical feasibility	Further supports the utility of PAP as a therapeutically relevant antigen in prostate cancer	Immune responses do not establish clinical benefit; the randomized phase II primary endpoint was not significantly improved.	[80,85]
Discovery of TM-PAP and Membrane-Related Studies	Studies of Splice Variants, Membrane Localization, and Intracellular Trafficking	Characterized transmembrane PAP with an extracellular domain and its membrane–endosome/lysosome trafficking	Provides a molecular basis for membrane-accessible PAP as a recognizable target	Provides a molecular basis for membrane-associated PAP targeting	[49,59]
Platforms Including TM-PAP-Targeted Antibodies, ADCs, and CAR-T Cells	3D8 antibody/ADC, fully human antibodies, PAP-targeted CAR-T, etc.	Tumor targeting, cellular expansion, or antitumor activity observed in vitro or in animal models	Evidence from different technology platforms supports the accessibility of membrane-associated PAP to drugs	Extends PAP targeting across additional therapeutic platforms.	[55,67,93]
High-Affinity OncoACP3 Ligand Screening	DNA-Encoded Chemical Libraries	Screening yielded the high-affinity ACP3 ligand OncoACP3	Extended ACP3 research to a small-molecule targeting platform that can be modularly conjugated to imaging or therapeutic payloads	Introduces a high-affinity small-molecule ACP3-targeting platform.	[40,94]
[^177^Lu]Lu-OncoACP3-DOTAGA and OncoACP3-GlyPro-MMAE	Preclinical radioligand therapy and small-molecule drug conjugation	Achieved selective tumor delivery and showed antitumor activity in ACP3-associated tumor models	Provides a preclinical basis for using ACP3 small-molecule ligands to deliver radionuclide and cytotoxic therapeutic payloads	Preclinical proof of payload delivery in xenograft models; no human therapeutic efficacy or therapeutic dosimetry established.	[40]
First-in-Human PET with [^68^Ga]Ga-OncoACP3-DOTA	25 patients with prostate cancer; retrospective matched comparison with [^18^F]PSMA-1007	Visualized lesions in localized and metastatic prostate cancer; overall lesion SUVmax did not differ significantly from that of the PSMA tracer, and normal-organ distribution differed	Provides preliminary evidence that ACP3-targeted PET can visualize lesions in humans and be evaluated in paired comparisons with PSMA	Retrospective, small cohort; the exploratory composite criterion does not establish diagnostic superiority.	[41]
Dynamic PET, Pharmacokinetics, and Diagnostic Dosimetry	Dynamic or multiple-time-point imaging in 7 patients with mCRPC	Described increasing tumor uptake, normal-tissue clearance, dual renal and hepatobiliary clearance pathways, and diagnostic radiation dose	Adds information on the time–activity behavior, biodistribution, and diagnostic dosimetry of ACP3-targeted PET in humans	Small diagnostic cohort; cannot establish absorbed doses or safety for therapeutic constructs.	[42]
Prospective phase I imaging study (Sollini et al.)	20 patients; prospective study; [^68^Ga]Ga-OncoACP3	PET positive in 17/20 patients; no tracer-related adverse events reported.	Early prospective human imaging and diagnostic-safety evidence.	Small cohort and selective histopathological verification; no established diagnostic superiority or therapeutic benefit.	[95]

## 4. Modern Evidence for ACP3 Theranostics

### 4.1. OncoACP3 Ligand Discovery and Preclinical Payload Delivery

Georgiev et al. screened 6,724,828 compounds from two DNA-encoded libraries against recombinant human PAP, the protein encoded by ACP3, and identified ProX1-(SS), subsequently termed OncoACP3. The ligand was initially identified by screening against purified recombinant PAP. Subsequent cell-based experiments showed that fluorescent ProX1-(SS) selectively bound membrane-associated PAP on PAP-expressing cells, with no detectable internalization under the reported confocal microscopy conditions. ProX1-(SS)-DOTAGA inhibited PAP with an IC_50_ of 0.30 nM. Radiolabeled OncoACP3 achieved high and selective uptake in PAP-expressing xenografts, with a tumor residence time of approximately 72 h in HT1080.hACP3 tumors and more than 72 h in PC3.hACP3 tumors, and a tumor-to-blood ratio exceeding 148 at 2 h. A single 20-MBq dose per mouse (1000 MBq/kg) of [^177^Lu]Lu-OncoACP3-DOTAGA produced complete responses in all four treated HT1080.hACP3 xenografts, while the cleavable OncoACP3-GlyPro-MMAE construct achieved tumor-selective MMAE deposition and antitumor activity [40]. These findings established OncoACP3 as a modular platform for radionuclide and cytotoxic payload delivery and provided the preclinical basis for subsequent human imaging studies [40,41,96].

The preclinical program established high-affinity recognition, target-selective tumor accumulation, sustained retention, and modular delivery of radionuclide and cytotoxic payloads. Translation can now optimize solubility, plasma stability, nonspecific binding, clearance, conjugation chemistry, and tumor retention and determine which ligand-accessible PAP forms drive binding in patients. Principles from small-molecule and antibody–drug conjugate development provide a framework for linker and payload-release optimization, with ACP3-specific cell, tissue, and in vivo assays used to confirm target dependence [60,61,97,98].

Radionuclide and MMAE constructs follow distinct pharmacological principles. The ^177^Lu construct can cross-irradiate adjacent cells through β radiation and is therefore less dependent on uniform target expression at the single-cell level, whereas the MMAE construct depends more strongly on linker stability, tumor-localized payload release, and uptake of released drug [40,60,61]. Accordingly, radionuclide and MMAE constructs should proceed through payload-specific optimization of efficacy, dosimetry or systemic exposure, and safety.

Beyond OncoACP3, recent studies have reported TM-PAP-directed monoclonal antibodies, antibody conjugates, fully human antibody scaffolds, and PAP-targeted chimeric antigen receptor T (CAR-T) cells. Kirschenbaum et al. reported that the 3D8 antibody and its ADC construct reduced tumor volume and prolonged animal survival in PAP-expressing VCaP xenograft models [55]; Comacchio et al. developed the fully human EKA4 antibody, which demonstrated tumor-targeting capability in in vivo biodistribution studies [67]; and Cao et al. reported PAP-coupled CAR-T activity with cellular expansion and antitumor effects in vitro and in animal models [93]. Across distinct technology platforms, these studies establish drug access to membrane-associated PAP and broaden the translational pipeline beyond OncoACP3. Cross-platform competition and tissue-binding studies can now determine how the PAP pools recognized by antibody, CAR-T, and OncoACP3 platforms overlap in vivo.

### 4.2. First-in-Human Pet with [^68^Ga]Ga-OncoACP3-DOTA

Backhaus et al. retrospectively compared paired [^68^Ga]Ga-OncoACP3-DOTA and [^18^F]PSMA-1007 PET examinations in 25 patients with prostate cancer [41]. After Bonferroni correction, no significant difference in SUVmax was observed between the tracers within any of the four analyzed lesion categories. Under the study-defined patient-level composite criterion of more than 10% additional lesions or more than 50% higher mean lesion SUVmax, ACP3-targeted PET was favored in 11 of 25 paired examinations, PSMA PET in eight, and neither tracer in six. This exploratory classification combines lesion detection and uptake intensity and should not be interpreted as evidence of superior diagnostic accuracy. Management changes were reported in three of six patients with biochemical recurrence and two of 12 patients with metastatic disease without previous [^177^Lu]Lu-PSMA-617 treatment; no adverse events were reported [41]. These observations suggest potential complementary information but do not establish improved clinical outcomes. Prospective paired studies should determine whether ACP3-targeted PET identifies verified, clinically relevant lesions not adequately characterized by PSMA imaging and whether this additional information leads to appropriate management changes or improved patient outcomes.

In the paired study, [^68^Ga]Ga-OncoACP3-DOTA showed lower salivary-gland and kidney uptake than [^18^F]PSMA-1007, indicating a different normal-organ uptake profile for these two diagnostic tracers [41]. The subsequent dynamic study identified dual renal and hepatobiliary clearance and comparatively high diagnostic absorbed-dose coefficients in red marrow, kidneys, and liver. Together, these findings define the organs and time–activity measurements to prioritize in construct-specific therapeutic dosimetry [37,42].

### 4.3. Dynamic Pharmacokinetics and Diagnostic Dosimetry

Brosch-Lenz et al. conducted dynamic or multiple-time-point PET in 7 patients with mCRPC and showed that [^68^Ga]Ga-OncoACP3-DOTA uptake decreased over time in most normal organs and was cleared through both renal and hepatobiliary pathways; by contrast, uptake in bone and lymph node metastases and tumor-to-background ratios (TBRs) increased over time. Kinetic analysis showed that an irreversible two-tissue-compartment model adequately described the time–activity behavior of metastatic lesions. The mean effective dose was 0.0192 mSv/MBq; for a standard injection of 2.5 MBq/kg, this corresponded to approximately 3.50 mSv; absorbed doses were relatively high in the bone marrow, kidneys, and liver, whereas the salivary gland dose was low [42].

The observed increase in tumor-to-background contrast over time suggests that imaging time is an important protocol variable for [^68^Ga]Ga-OncoACP3-DOTA. Optimization should balance lesion contrast, normal-tissue clearance, workflow feasibility, and the physical decay of ^68^Ga [99,100].

The human [^68^Ga]Ga-OncoACP3-DOTA study provides a starting biodistribution framework for dedicated therapeutic dosimetry studies [42]. However, its diagnostic absorbed-dose estimates cannot establish organ absorbed doses or therapeutic safety for [^177^Lu]Lu-OncoACP3-DOTAGA or future ^225^Ac-labeled OncoACP3 constructs. Differences in radionuclide, chelator, linker, clearance, and tissue residence time preclude assuming equivalent pharmacokinetics or organ exposure across these constructs. Each therapeutic construct therefore requires dedicated serial measurements of tumor and normal-organ activity, construct-specific absorbed-dose estimation, and clinical toxicity assessment. Established radioligand dosimetry recommendations provide a methodological reference, rather than ACP3-specific therapeutic evidence [37].

### 4.4. From Early Human Evidence to Confirmatory Studies

**Figure 3:**
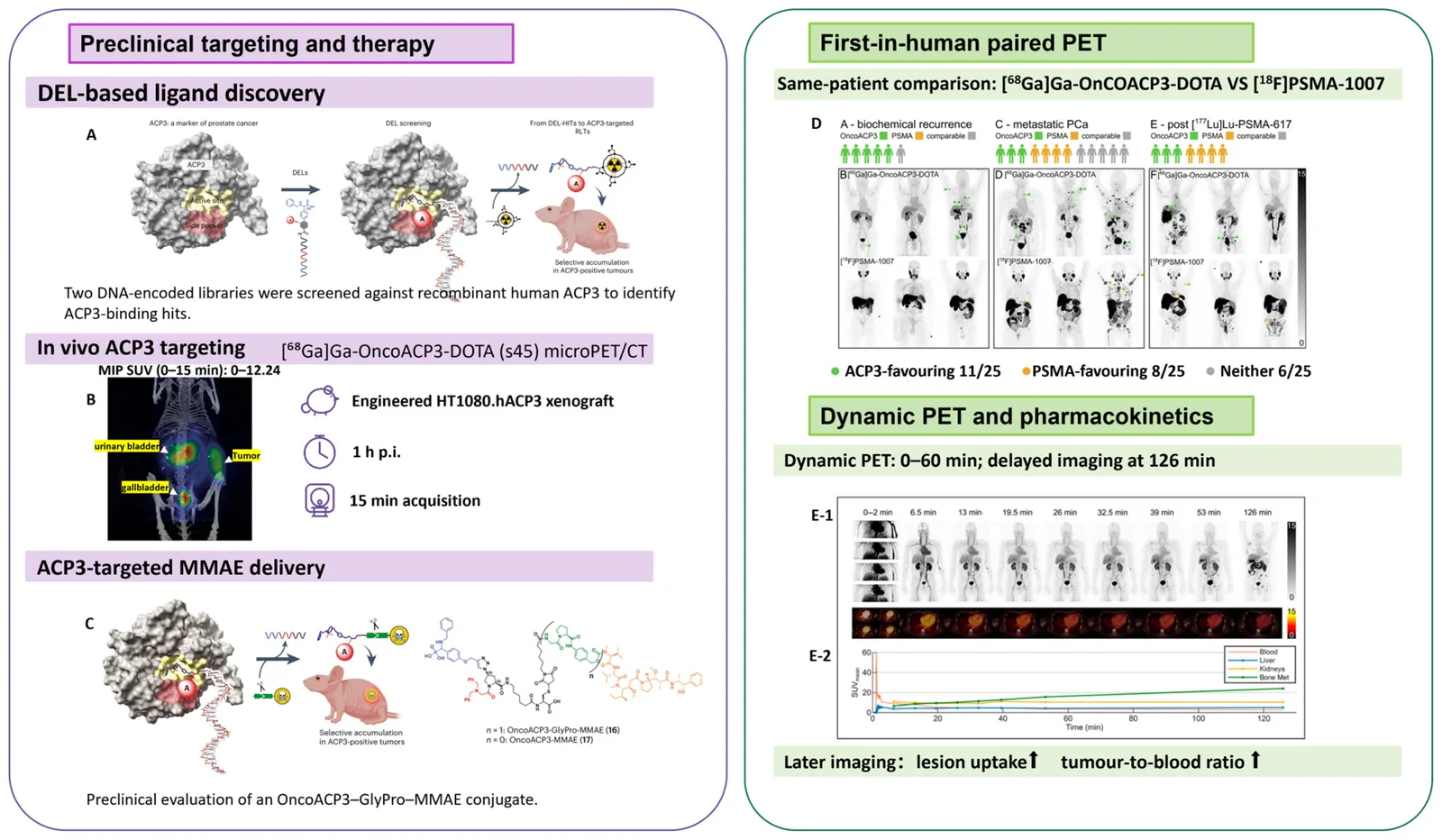
Preclinical development and early clinical translation of ACP3-targeted imaging and therapeutic strategies. (**A**) Screening of two DNA-encoded libraries against recombinant human PAP, the protein encoded by ACP3, and development of selected hits as ACP3-targeted radioligands. (**B**) [^68^Ga]Ga-OncoACP3-DOTA (s45) microPET/CT in an engineered HT1080.hACP3 xenograft model at 1 h post-injection, showing tumour uptake with activity in the gallbladder and urinary bladder. (**C**) Development of OncoACP3 conjugates for tumour-selective delivery of monomethyl auristatin E (MMAE). (**D**) Representative same-patient comparisons of [^68^Ga]Ga-OncoACP3-DOTA and [^18^F]PSMA-1007 PET in patients with biochemical recurrence, metastatic prostate cancer, or previous [^177^Lu]Lu-PSMA-617 treatment. Under the original study-defined composite criterion of more than 10% additional lesions or more than 50% higher mean lesion SUVmax, ACP3-targeted PET was favored in 11/25 paired examinations, PSMA PET in 8/25, and neither tracer in 6/25. These exploratory classifications do not establish diagnostic superiority. The internal A–F labels in panel D are retained from the source figure [41] and are distinct from the main panel labels of this composite figure. (**E-1**) Representative dynamic [^68^Ga]Ga-OncoACP3-DOTA PET images acquired over 0–60 min, with delayed imaging at 126 min. (**E-2**) Corresponding time–activity curves showing increasing lesion uptake and tumour-to-blood contrast at later time points. ACP3, acid phosphatase 3; DEL, DNA-encoded library; MIP, maximum-intensity projection; MMAE, monomethyl auristatin E; PCa, prostate cancer; PET, positron emission tomography; PET/CT, positron emission tomography/computed tomography; p.i., post-injection; PSMA, prostate-specific membrane antigen; RLT, radioligand therapy; SUV, standardized uptake value; SUVmean, mean standardized uptake value. Panels (**A**–**C**) incorporate material adapted or reproduced from [40], with permission from Springer Nature. Panel (**D**) was adapted from [41] under the Creative Commons Attribution 4.0 International licence (https://creativecommons.org/licenses/by/4.0/, accessed on 26 October 2026); the original material was cropped and reformatted. Panels (**E-1**,**E-2**) were reproduced and reformatted from [42] in accordance with the JNM permission policy. This research was originally published in JNM: Brosch-Lenz J, Büther F, Oehler S et al. Dynamics, Pharmacokinetics, and Dosimetry of First-in-Human [^68^Ga]Ga-OncoACP3-DOTA PET in Patients with Prostate Cancer. *J Nucl Med.* 2026;67:1079–1085. © SNMMI. https://doi.org/10.2967/jnumed.125.271933. Material in panels (**A**–**C**) and (**E-1**,**E-2**) is excluded from the CC BY 4.0 licence applied to this article and remains subject to the respective copyright holders’ reuse terms.

Early human investigations include a paired PET study demonstrating lesion visualization and comparison with PSMA, and a dynamic PET study defining time–activity behavior, tumor retention, clearance, and diagnostic dosimetry [41,42]. Together with high-affinity ligand discovery and preclinical payload delivery, these studies establish a continuous translational evidence chain from target engagement to human imaging and pharmacokinetics (Figure 3) [40,41,42]. This foundation supports the next phase of clinical development: construct-specific therapeutic pharmacokinetics, dosimetry, safety, and efficacy studies.

Sollini et al. subsequently reported a prospective phase I study of [^68^Ga]Ga-OncoACP3 in 20 patients. PET was positive in 17 of 20 patients, and no tracer-related adverse events were reported [95]. This provides prospective imaging and diagnostic-safety evidence, while the small cohort and selective histopathological verification limit generalization; diagnostic superiority and therapeutic benefit remain unestablished.

## 5. ACP3 and PSMA: Potential Complementarity and Its Evidence Base

### 5.1. Target Biology, Expression Profiles, and Maturity of Evidence

The principal distinction between PSMA and ACP3-targeted platforms is the maturity of their evidence. PSMA is supported by prospective imaging studies, standardized interpretation, and randomized treatment trials [19,23,24,25,101,102]. For ACP3-targeted ligand development, the evidence includes preclinical payload delivery [40] and early human imaging and diagnostic dosimetry [41,42,95]. The clinical benefit of PAP-directed immunotherapy [9] represents a separate mechanism and does not validate direct ligand targeting. Accordingly, differences in uptake from an exploratory PET comparison should not be interpreted as differences in overall diagnostic or therapeutic value. The relevant comparison is whether ACP3 supplies clinically meaningful information or target coverage that is insufficiently provided by PSMA in a defined patient population.

Target definition also differs. PSMA is a type II transmembrane protein [103,104], whereas PAP occurs in secretory, intracellular, and transmembrane compartments [54,59,63]. Early OncoACP3 imaging demonstrates that a PAP-associated target pool can be visualized in patients [41], but does not fully identify its molecular form or location. Same-lesion studies integrating PAP localization, ex vivo ligand binding, and PET uptake are needed to relate total protein expression to the ligand-accessible pool.

Expression heterogeneity must be evaluated directly rather than inferred from separate tissue cohorts. In particular, reduced PAP positivity in small-cell neuroendocrine carcinoma [69] cautions against assuming that ACP3 targeting will compensate for PSMA loss in all poorly differentiated lesions. Paired imaging and, where appropriate, tissue validation should determine whether the two targets provide complementary coverage within the same patient. The biology of discordant lesions is therefore more informative for this question than the overall positivity rate of either marker.

### 5.2. Normal-Organ Distribution and Therapeutic Index

The paired human study found lower salivary-gland and kidney uptake with [^68^Ga]Ga-OncoACP3-DOTA than with [^18^F]PSMA-1007 [41]. This is evidence of a different distribution relative to the specific comparator, not proof of lower toxicity or a general advantage over all PSMA ligands. The dynamic study further characterized mixed renal and hepatobiliary clearance and diagnostic organ-dose coefficients [42]. These observations identify normal tissues requiring attention during development; the quantitative findings are presented in Section 4.3.

Therapeutic index depends on tumor and normal-organ absorbed doses, lesion coverage, retention, and the properties of the individual construct. Consequently, human gallium-68 diagnostic data cannot establish the therapeutic dosimetry of [^177^Lu]Lu-OncoACP3-DOTAGA or future α-emitting constructs. The current evidence supports dedicated therapeutic evaluation, with tumor uptake considered alongside critical-organ exposure and repeated-administration safety. Section 6.3 outlines the construct-specific requirements. A favorable diagnostic image or low uptake in one organ alone is insufficient to establish a therapeutic advantage.

### 5.3. Clinical Scenarios for Testing ACP3–PSMA Complementarity

Potential clinical value should be tested in predefined settings, including active disease with insufficient PSMA uptake, marked interlesional heterogeneity, or progression after PSMA-targeted treatment. These are candidate research populations, not established indications for ACP3-directed care. Studies should determine whether ACP3-targeted PET detects additional lesions confirmed by histology or follow-up and whether those findings change an actionable decision, such as biopsy-site selection, radiotherapy planning, or trial enrollment. An observed management change should be distinguished from subsequent improvement in clinical outcomes. The prospective validation requirements are detailed in Section 6.4.

Longitudinal paired imaging could additionally assess whether progressive lesions retain ligand-accessible PAP as PSMA uptake changes. Its value must be weighed against additional examinations, radiation exposure, and resource use. Sequential or combined treatment remains a separate research question requiring adequate safety and dosimetry data for the individual therapeutic constructs. Table 3 summarizes the evidence supporting the two targets and the boundaries of the proposed complementarity.

## 6. Translational Challenges and Clinical Development Pathway

### 6.1. Establishing A Ligand-Specific Target Definition and Companion Diagnostic

Before ACP3-targeted strategies enter clinical validation, the PAP form predominantly recognized by OncoACP3 in patient tumors and its accessible compartment need to be further clarified. PAP occurs in different forms, including secretory, intracellular, and transmembrane forms, whereas the correspondence of conventional ACP3 RNA expression or PAP immunohistochemistry with in vivo ACP3-targeted PET uptake using OncoACP3 has not yet been established. Studies integrating ligand binding, PAP form-specific expression, and spatial localization in tissue samples with lesion-level ACP3-targeted PET uptake could clarify which PAP pools are accessible to OncoACP3 and how protein abundance and localization relate to imaging findings.

Reproducible associations between tissue testing and imaging metrics also need to be established. Serum PAP has shown associations with disease burden and prognosis in historical studies [64,65,66,76,115], but its circulating concentration does not establish the amount, localization, or accessibility of PAP available for ligand binding within individual lesions. ACP3-based patient stratification is therefore more likely to rely on complementary information from tissue and whole-body molecular imaging, with subsequent studies clarifying the relationships among these metrics.

### 6.2. Standardizing PET Protocols and Positivity Thresholds

As ACP3-targeted PET advances from early feasibility studies to multicenter validation, the reproducibility of parameters such as injected activity, ligand mass, imaging time, reconstruction method, SUV, and tumor-to-background ratio needs to be further clarified [41,42,95]. Paired evaluation with PSMA PET and, when necessary, FDG PET at the same-patient level could be used to characterize lesion concordance, incremental detection [116], and uptake patterns at different anatomical sites, with true lesion status confirmed by histology or composite follow-up.

The diagnostic positivity threshold and the treatment-selection threshold may not be the same. The former focuses mainly on lesion detection, whereas the latter also involves whole-body lesion coverage, uptake heterogeneity, and normal-organ exposure. Therefore, establishment of an ACP3-targeted PET threshold requires consideration of the relationships between continuous uptake metrics and tissue PAP expression, lesion dose, and clinical outcomes, with its reproducibility validated in independent cohorts. The effects of factors such as renal function, hepatic function, prior treatment, and imaging time on background clearance also need to be controlled in multicenter studies.

### 6.3. From Diagnostic Dosimetry to Therapeutic Dosimetry

For therapeutic development, serial tumor and normal-organ time–activity measurements should be obtained separately for [^177^Lu]Lu-OncoACP3-DOTAGA and any future ^225^Ac-labeled OncoACP3 construct. Red marrow, kidneys, and liver warrant particular attention based on the diagnostic biodistribution findings [42], without assuming that they will necessarily be the dose-limiting organs for therapeutic administration. Construct-specific absorbed-dose estimates should be evaluated alongside clinical toxicity, including cumulative effects during repeated administration, to assess the therapeutic window.

Different therapeutic payloads also have different dosimetric and pharmacological requirements. The β radiation of ^177^Lu has a finite tissue range and produces a degree of cross-fire irradiation in the setting of heterogeneous target distribution within lesions; the short range of α radionuclides makes microscopic target distribution, cellular binding, and retention more important. Cytotoxic payloads such as MMAE are also influenced by linker stability, tumor-localized payload release, uptake of the released drug by tumor cells, and other pharmacological properties [40,60,61]. Therefore, evaluation of therapeutic constructs needs to consider tumor uptake, critical-organ doses, lesion heterogeneity, and the pharmacological characteristics of the payload itself.

### 6.4. Patient Selection and Clinical Validation

Patient selection for ACP3 clinical studies should correspond to the clinical question that this study is intended to address. The settings most worthy of validation at the present stage include patients with low or negative PSMA uptake but persistent active disease, patients who develop selective progression after PSMA-targeted therapy, and patients with marked heterogeneity of PSMA expression between lesions. In these populations, whether ACP3-targeted PET can detect validated additional lesions and whether this information changes patient stratification or actionable treatment decisions are key questions in evaluating its complementary value.

After therapeutic ACP3 constructs enter human studies, early clinical evaluation will also need to incorporate individualized dosimetry, critical-organ exposure, whole-body lesion coverage, and preliminary antitumor activity [117,118]. As evidence accumulates, subsequent studies may further evaluate patient-relevant outcomes such as radiographic progression-free survival, overall survival, symptoms, quality of life, and organ toxicity [119]. Sequential or combined use of ACP3 and PSMA is also more appropriately validated after the dosimetry and safety of single-target constructs have been sufficiently established.

### 6.5. Multicenter Implementation and Reproducibility of Results

After the ACP3 theranostic platform enters multicenter studies, the reproducibility of radiochemical preparation, image acquisition, quantitative analysis, and dosimetry methods will directly affect the interpretation of study results. After OncoACP3 is conjugated to different chelators, radiometals, or therapeutic payloads, its chemical identity, stability, and biological activity need to be confirmed [40,41,42,60]; key technical parameters that affect in vivo distribution and quantitative results also need to be harmonized across centers as much as possible.

Standardized imaging time, interpretation criteria, and dosimetric analysis will determine whether early results can be reproduced across centers [120,121]. At the present stage, clinical implementation should prioritize consistency in core quality metrics, imaging quantification, and patient-safety evaluation, thereby creating a comparable data basis for confirmatory studies.

Overall, the progression of ACP3 from early human imaging to further clinical translation primarily involves five interrelated issues: the PAP form actually recognized by the ligand needs to be clarified, PET metrics need to be standardized, independent dosimetry needs to be established for therapeutic constructs, clinical studies need to validate the target patient populations and patient-relevant benefits, and multicenter studies need to ensure the reproducibility of preparation and quantitative results. These issues define the principal validation steps required for the current ACP3 evidence chain to progress from “capable of imaging in humans” to “capable of guiding treatment”.

## 7. Limitations of the Current Evidence and This Review

Modern ACP3 theranostic evidence is concentrated on the OncoACP3 platform: a 25-patient paired PET study and a 7-patient dynamic pharmacokinetic and dosimetry study establish early human imaging feasibility, biodistribution, and kinetic benchmarks [41,42]. A subsequent prospective phase I study in 20 patients adds early prospective imaging and diagnostic-safety evidence [95]. Larger independent prospective multicenter cohorts remain necessary for defining diagnostic performance, positivity thresholds, and appropriate patient populations across disease stages and treatment histories. [^177^Lu]Lu-OncoACP3-DOTAGA and OncoACP3-GlyPro-MMAE have established preclinical proof of concept [40], while emerging antibody and CAR-T platforms broaden the preclinical pipeline [55,67,93]. These results provide a basis for first-in-human evaluation of therapeutic pharmacokinetics, tumor and organ absorbed doses, multicycle safety, and antitumor activity. Diagnostic biodistribution should serve as a starting benchmark for these construct-specific therapeutic studies.

Existing evidence concerning ACP3 and its encoded protein PAP also spans multiple distinct biological levels. Serum PAP primarily reflects secretion-related burden and disease status; PAP immunohistochemistry reflects tissue origin and protein expression; intracellular PAP and TM-PAP have different subcellular localizations and functions, whereas ACP3-targeted PET uptake provides an in vivo measure influenced by ligand-accessible PAP, tracer delivery, retention, and clearance; treatment response is further influenced by factors including whole-body lesion coverage, therapeutic payload delivery, lesion absorbed dose, and critical-organ dose. Therefore, serum PAP levels, tissue PAP expression, membrane-accessible PAP, ACP3-targeted PET uptake, and response to ACP3-targeted therapy should be regarded as interrelated levels of evidence that require separate validation. In the future, paired studies at the same-patient or same-lesion level will be required to clarify the correspondence among these metrics and establish a validation framework that links tissue protein expression, molecular imaging, and treatment response [9,40,41,42,54,59,69].

This article is a narrative review and did not include a systematic evaluation, formal quantification of bias, or meta-analysis; the included studies are heterogeneous in patient selection, testing methods, tracers, experimental models, and study endpoints, and are therefore not suitable for rigorous quantitative cross-study comparisons. Some recent evidence is derived from commentaries, reviews, or early preclinical studies, whose primary role is to supplement the research background and define potential research directions; key clinical judgments should still be based on original human studies. Given that ACP3 theranostic translation remains a rapidly developing field, subsequent studies of human imaging, therapeutic constructs, and independent validation may further change the current evidence landscape.

## 8. Conclusions

PAP has progressed from a historical serum marker and pathological lineage marker to an immunotherapeutic antigen and, more recently, a ligand-accessible target for molecular imaging and payload delivery. The OncoACP3 program provides a coherent translational sequence: high-affinity ligand discovery, target-dependent preclinical tumor localization, preclinical radionuclide and cytotoxic efficacy, first-in-human PET, and diagnostic pharmacokinetics and dosimetry.

The evidence currently supports ACP3 as an emerging complementary target, not a replacement for PSMA. Early human imaging suggests partially discordant lesion information and a different normal-organ distribution, but diagnostic superiority, therapeutic dosimetry, and treatment efficacy in humans have not been established.

The next phase should resolve four linked questions: which PAP pool is accessible to each ligand, how PET positivity should be defined, whether therapeutic constructs deliver a favorable tumor-to-organ dose, and which patients gain actionable information or treatment benefit beyond established PSMA pathways. Answering these questions prospectively will determine whether ACP3 becomes a clinically useful theranostic target or remains a promising biological and imaging marker.

## Figures and Tables

**Table 3:** Evidence base, current positioning, and boundaries of evidence for PSMA and ACP3 as theranostic targets in prostate cancer.

Comparison Dimension	PSMA	ACP3	Current Interpretation	Reference(s)
Molecular forms and membrane accessibility	A well-defined type II transmembrane protein with an extracellular domain that can be recognized by small molecules or antibodies	PAP occurs in different forms, including secretory, intracellular, and transmembrane forms; the PAP molecular pool predominantly recognized by OncoACP3 in humans has not yet been fully elucidated	The PSMA target definition and membrane accessibility are relatively well defined; for ACP3, the correspondence among ligand binding, PAP protein expression in tissue, and in vivo ACP3-targeted imaging still needs to be established	PSMA [16,103,105]; ACP3 [40,41,49,59,63]
Prostate cancer-associated expression	Expressed in most prostate adenocarcinomas, but marked heterogeneity may exist between patients, between lesions, and after treatment	PAP has a strong association with the prostate; PAP expression is reduced in poorly differentiated tumors and some neuroendocrine tumors	Both targets exhibit heterogeneity related to disease stage and differentiation; their lesion-level distributions may not fully overlap.	PSMA [11,12,16,28,106]; ACP3 [68,69,70]
PET imaging evidence	Multiple prospective, multicenter, and standardized studies have been conducted, and it is widely used for staging, localization of biochemical recurrence, and treatment selection	Currently derived mainly from a first-in-human paired PET study of 25 patients and a dynamic/multiple-time-point study of 7 patients, together with a subsequent prospective phase I study in 20 patients	ACP3-targeted PET has demonstrated the feasibility of human imaging but remains at an early clinical stage.	PSMA [19,20,21,22,101,102,107,108]; ACP3 [41,42,95]
Therapeutic evidence	[^177^Lu]Lu-PSMA-617 and other agents have been evaluated in multiple randomized clinical trials and have received regulatory approvals; clinical application has further expanded from mCRPC to earlier stages of PSMA-positive metastatic disease	[^177^Lu]Lu-OncoACP3-DOTAGA, MMAE constructs, and platforms such as antibodies and CAR-T are currently supported mainly by preclinical evidence	PSMA has randomized clinical evidence; ACP3 therapeutic evidence is currently preclinical.	PSMA [23,24,25,26,27,38]; ACP3 [40,55,67,93]
Normal-organ distribution	Marked physiological uptake occurs in the salivary glands, lacrimal glands, kidneys, and portions of the intestine	The paired diagnostic study showed lower salivary-gland and kidney uptake than [^18^F]PSMA-1007; TM-PAP has also been reported to be present in multiple nonprostatic tissues	Early human imaging suggests differences in normal-organ distribution between ACP3- and PSMA-targeted tracers; therapeutic constructs require separate evaluation	PSMA [35,37,101,109,110]; ACP3 [41,42,59]
Pharmacokinetics and therapeutic dosimetry	Substantial experience has been accumulated regarding human therapeutic dosimetry, administered activity, number of cycles, and critical-organ toxicity	Human diagnostic dosimetry has been reported for [^68^Ga]Ga-OncoACP3-DOTA; these measurements do not establish tumor or organ absorbed doses for therapeutic OncoACP3 constructs.	Diagnostic biodistribution provides an initial framework; therapeutic absorbed doses and safety require construct-specific assessment.	PSMA [37,38,111]; ACP3 [42]
Lesion heterogeneity and potential complementarity	Some lesions may exhibit low or negative PSMA uptake, and target expression may also change under treatment pressure	The first-in-human study suggested that ACP3 and PSMA tracers may provide incompletely overlapping information in some patients and lesions	Early paired imaging suggests partially nonoverlapping lesion information.	PSMA [11,12,16,28,29,30,106]; ACP3 [41]
Current clinical positioning	An established target for molecular imaging and radioligand therapy in prostate cancer	A candidate complementary target at an early stage of human theranostic translation	The potential incremental value of ACP3 can be further validated in predefined populations with low PSMA uptake, lesion heterogeneity, or progression after PSMA-targeted therapy	[19,23,25,41,42,95,101,112,113,114]

## Data Availability

No new data were created or analyzed in this study. Data sharing is not applicable to this article.
